# Comparative analysis of molecular and physiological traits between perennial *Arabis alpina* Pajares and annual *Arabidopsis thaliana* Sy-0

**DOI:** 10.1038/s41598-017-13606-7

**Published:** 2017-10-17

**Authors:** Jong-Yoon Park, Hoyeun Kim, Ilha Lee

**Affiliations:** Laboratory of Plant Developmental Genetics, School of Biological Sciences, Plant Genomics & Breeding Institute, Seoul National University, Seoul, 08826 Korea

## Abstract

Annual plants complete life cycle in a year while perennial plants maintain growth for several years. *Arabis alpina*, a polycarpic perennial, is a close relative of monocarpic annual Arabidopsis. Pajares is an accession of *A*. *alpina* requiring vernalization, a long-term cold for flowering. Arabidopsis shows holistic flowering whereas Pajares shows idiographic flowering, producing axillary branches under variable developmental phases from juvenile, adult vegetative to reproductive phases. To understand the molecular mechanism behind diverse phases of axillary branches, we analyzed the levels of primary miR156 expressions because miR156-SPL module is a key regulator for developmental phase transition. We found that in Pajares, miR156 levels were highly variable among the axillary branches, which causes differential sensitivity to vernalization. Thus, the axillary branches expressing high levels of miR156 remain in juvenile phase even after vernalization, whereas the axillary branches expressing low levels of miR156 produce flowers after vernalization. In contrast, every axillary branches of Arabidopsis winter annual Sy-0 expressed similar levels of miR156 and synchronously responded to vernalization, which causes holistic flowering. Therefore, we suggest that variable miR156 expression levels and the resulting differential response to vernalization among axillary branches are distinctive features determining polycarpic perenniality of *A*. *alpina* Pajares.

## Introduction

Plant kingdom is largely divided into semelparous monocarpic and iteroparous polycarpic plants depending on the life cycle strategies. The monocarpic species include all annuals and some perennials such as bamboo. They show holistic senescence following massive flowering at once to maximize number of offsprings. Arabidopsis is a representative annual model plant, which lives a single growing season and complete their life cycle within a year. In Arabidopsis, rapid cycling accessions such as Columbia and Landsberg *erecta* execute early-flowering whereas winter annual accessions require prolonged cold for 4~8 weeks to accelerate flowering. Some perennial species, such as bamboo, maintain vegetative phase for many years before flowering and then undergo holistic senescence to die^1^. On the other hand, most polycarpic species repeat several cycles of reproduction (iteroparous reproduction) and ensure long-term survival. Polycarpic perennials prolong their lives by maintaining vegetative shoots and/or producing new vegetative shoots even after transition to reproductive phase^1–3^. Polycarpic perennial *Arabis alpina* (Alpine rock-cress) is a close relative to Arabidopsis which belongs to *Brassicaceae* family. *Arabis alpina* is widely spread in mountainous areas of Europe, North and East Africa, Central and East Asia, and North America^4^. Genome size of *Arabis alpina* is 392 Mb and it is self-compatible^5^. Pajares is a vernalization requiring accession among more than 140 accessions of *Arabis alpina*^6–10^. A life cycle of plants usually consists of discrete developmental phases. During germination, seedlings sprout from seeds through embryonic to post-embryonic developmental transition. The seedlings increase their mass during vegetative phase. The vegetative phase is further divided into juvenile and adult phases, and plants acquire competence to flower through such changes. Before transition to reproductive phase, plants become competent to flower and eventually produce reproductive organs and seeds^11,12^. Plants show heteroblasty because the same plants have leaves with different morphological traits developed from juvenile to adult vegetative phases. The early rosette, juvenile leaves of Arabiodpsis are small and almost round in shape with smooth margins and long petioles, while adult rosette leaves are enlarged and elongated with visible leaf serrations. Leaf trichomes are detected on both abaxial and adaxial surfaces in adult vegetative leaves, but juvenile leaves have leaf trichomes only on adaxial side^13^. During plant development, many environmental and endogenous cues affect phase transition. The environmental signals such as light intensity, photoperiod, and ambient temperature as well as endogenous hormones and aging influence the timing of phase transition. A post-transcriptional regulatory module, microRNA156 - SQUAMOSA PROMOTER BINDING PROTEIN-LIKE (SPL), is highly conserved in various plant species and acts as an age-dependent timer regulating phase transitions^13–21^. The expression level of miR156 is higher at seedling stage and is gradually decreased according to age. A wide range of transgenic plant species including Arabidopsis, rice, maize, poplar hybrid tree overexpressing miR156 produce excessive number of juvenile leaves and flower extremely late^13,22^. Such reports suggest that miR156 has functions promoting juvenile phase and delaying developmental phase transition. In Arabidopsis, 11 of 17 genes encoding SPL transcription factors are revealed as targets of miR156, while direct upstream factors of miR156 remain to be discovered^12^. The SPL proteins redundantly act in developmental phase transitions from embryogenesis to reproductive phase^12,13,23–26^.

In recent decades, several genetic factors to regulate developmental phase transition have been studied in Arabidopsis. However, molecular studies in perennials are limited because of long life cycle, scarcity of genomic resources, and difficulties in handling. As regulation of flowering is particularly important in biomass and yield, researchers studying perennial plants have focused on the orthologs of Arabidopsis flowering genes^27–32^. To figure out a life strategy of perennial plants, we compared the molecular and physiological features of a perennial plant, *Arabis alpina* Pajares, and a close relative annual, *Arabidopsis thaliana* Sy-0. As a result, we observed that asynchronized expression of *pre-miR156*s in axillary branches of *A*. *alpina* results in the variable responsiveness to vernalization, thus the axillary branches incompetent to flower are remained as vegetative branches after winter cold. In contrast, winter annuals of *A*. *thaliana* showed synchronized expression of *pre-miR156* in all the axillary branches. Therefore, we propose that variable expression of miR156 in axillary branches confers polycarpic perenniality to *A*. *alpina*.

## Results

### In *Arabis alpina* Pajares, axillary branches undergoing different developmental phases coexist in the same plant

*A*. *alpina* Pajares has distinctive features compared to a close relative annual plant, *Arabidopsis thaliana*. For example, Pajares shows internode elongation and outgrowth of axillary branches from each node during vegetative phase whereas *A*. *thaliana* develops rosette leaves due to lack of internode elongation during vegetative growth. In addition, Pajares has to pass through at least 5 weeks of juvenile phase to respond to vernalization, a long-term winter cold for flowering, and absolutely requires more than 10 weeks of vernalization for flowering^33^. The most interesting feature of Pajares is that each axillary branch undergoes whole life cycle, from juvenile to adult, and reproductive phases, as like an individual plant. In a primary shoot, the four basalmost true leaves show juvenile features such as unexpanded leaves with smooth margins (Figure S1A and C). These four juvenile leaves are produced within about 3 weeks after germination (Figure S2A). Similarly, juvenile leaves are produced at the 3 basal nodes of axillary branches (Figure S1B and C). To compare the growth patterns of axillary branches with those of primary shoots, we measured shoot-length, number of true leaves every week and they showed similar growth rate (Figure S2A and B). For the analysis of growth patterns in axillary branches, we categorized each axillary branch depending on the developmental stage from S1 to S5 (description of developmental stage in Figure S2 and Table S1). The S1 branch is the latest and S5 branch is the oldest one we used for the analysis.

**Figure 1:**
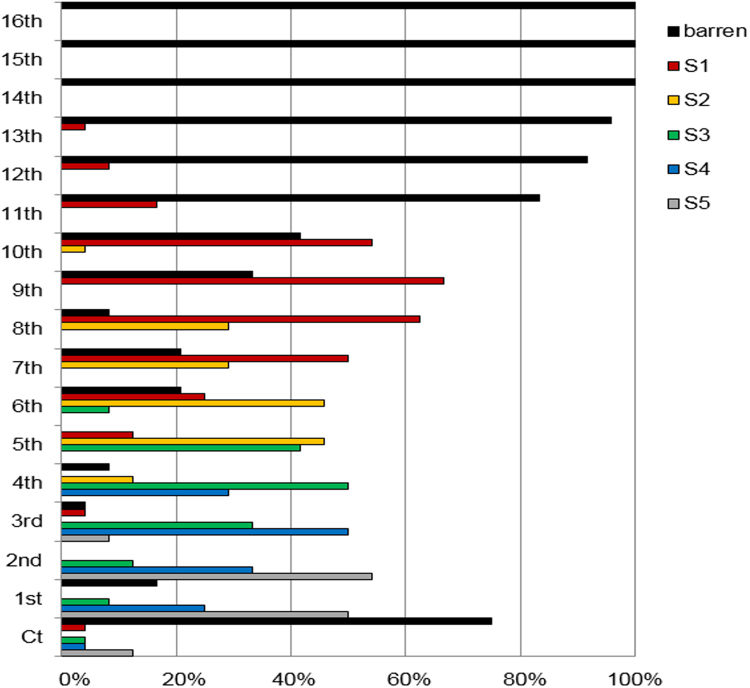
Proportion of axillary branches undergoing particular developmental stages at each node. Composition of axillary branches undergoing from S1 to S5 stages at each node of 8-week old plants, *A*. *alpina* Pajares. Y axis indicates position of each node counted from cotyledons (Ct). X axis indicates proportion of axillary branches at particular developmental stages. (n = 24).

An 8-week old Pajares, which is in vegetative phase before vernalization, has several axillary branches undergoing variable developmental stages from S1 to S5 and we labelled them as 8WS1 to 8WS5. The 8WS5 branches have been developed at the 4 basalmost nodes of primary shoot. To monitor branching order, proportion of axillary branches in each developmental stage from all node-positions from cotyledons to shoot apices was calculated using 24 plants of 8-week old Pajares (Fig. 1). In general, axillary branches in Pajares developed acropetal direction, albeit there were some exceptions at the two basalmost nodes. Most of the axillary branches in 8WS5 were found at the 1st and 2nd nodes from cotyledons. In contrast to this, the axillary branches in 8WS1 were mostly located near the shoot apices, at the 7th to 10th nodes from cotyledons. Therefore, our observation of Pajares growth strongly indicates that axillary branches generated from the same plant show asynchronous development with variable developmental stages from S1 to S5.

### MicroRNA156 expression levels in axillary shoot apices are variable depending on the developmental stages in *A*. *alpina* Pajares

**Figure 2:**
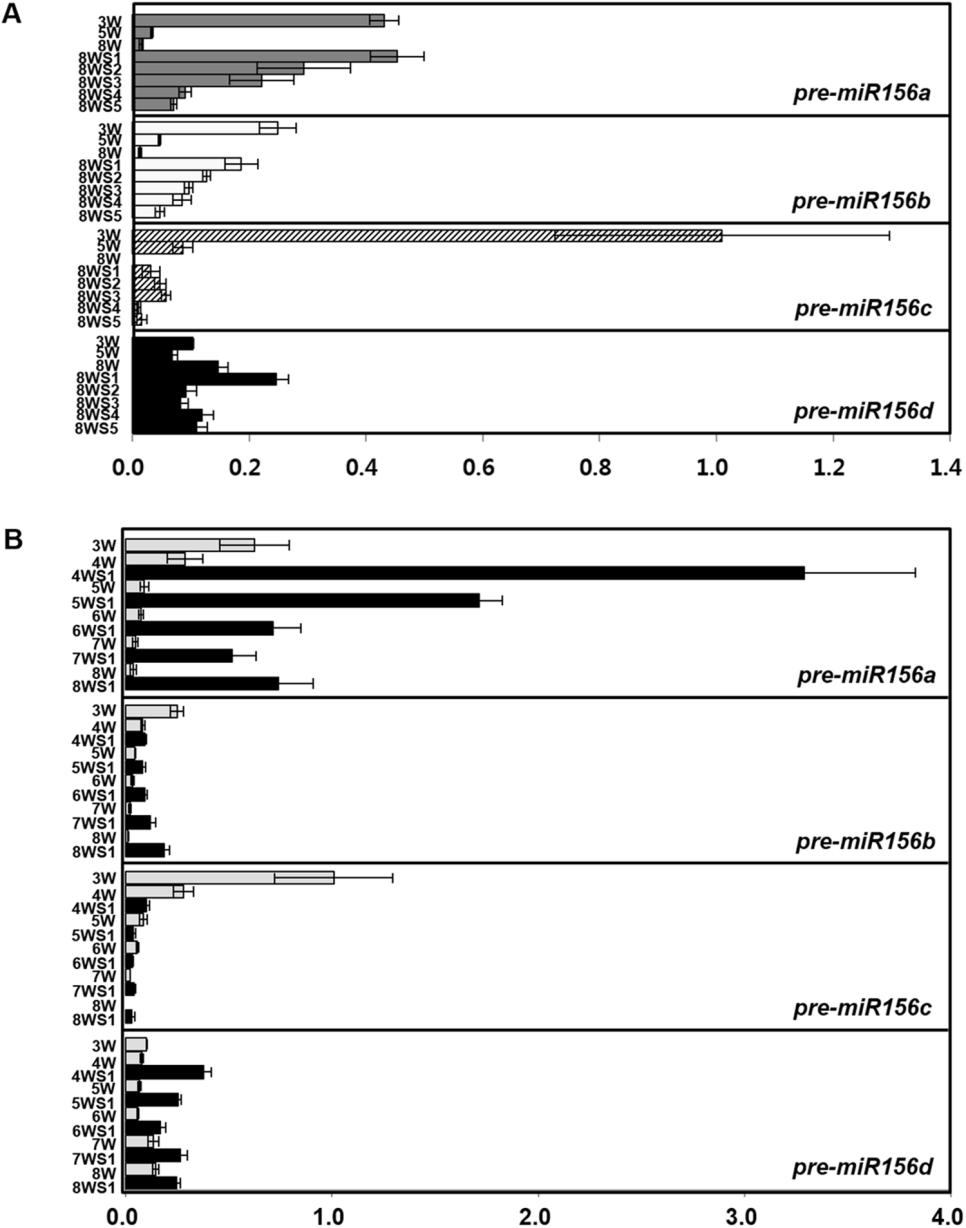
Expression levels of miR156 precursors in the primary and axillary shoot apices during vegetative growth of *A*. *alpina*. (**A**) The primary shoot apices of 3-week (3W), 5-week (5W), and 8-week (8W) old *A*. *alpina* were collected for the comparison of expression levels. The axillary shoot apices in each developmental stage from S1 to S5, labelled as 8WS1 to 8WS5, were collected from 8-week old plants and the expression levels were compared. Relative expression levels of primary *miR156a*, *b*, *c* and *d* were normalized to *PP2A* expression. (**B**) Expression levels of *pre-miR156a*, *b*, *c* and *d* in S1 shoot apices developed from 4-week to 8-week old plants were compared. Gray bars, miR156 precursor levels in the primary shoot apices from 3-week to 8-week old (3W~8W) plants. Black bars, miR156 precursor levels in the S1 axillary shoot apices from 4-week to 8-week old (4WS1~8WS1) plants.

Age-dependent decrease of miR156 level is conserved in diverse plant species^18^. To test if miR156 level is also decreased according to developmental progress in axillary branches of *A*. *alpina* Pajares, we cloned six homologs of miR156 precursors using *A*. *alpina* database^34^ and designated them as *pre-miR156a*, *b*, *c*, *d*, *f* and *g* depending on the phylogenic analysis and prediction of secondary structure (Figures S3 and S4). Since expression levels of miRNA precursors reflect mature miRNA levels in several plant species^17,25,26^, we analyzed quantities of six miR156 precursors by qRT-PCR, which amplifies the precursor sequences covering the target binding site (131 to 276 bp). In primary shoot apices, the relative expression levels of *pre-miR156a*, *b*, *c*, and *d* were gradually decreased upon aging, especially the expression levels of *pre-miR156a* and *c* were dramatically reduced from 2 to 3-week (Figure S5). Transcript levels of *pre-miR156f* and *g* were too low to compare even in young shoot apices. Next, we examined relative expression levels of miR156 precursors in the axillary shoot apices, which are in various developmental stages, developed from 8-week-old Pajares. For this analysis, we collectively harvested the axillary shoots in the same developmental stage (based on the size as shown in Figure S2) from all positions of nodes. The expression levels of *pre-miR156a* and *b* were gradually decreased according to progressive development from 8WS1 to 8WS5 stages. Levels of *pre*-*miR156c* and *d* in axillary branches were low and not strictly dependent on the development (Fig. 2A). Thus, Pajares shows asynchronous development of axillary branches undergoing variable stages from S1 to S5, which is manifested in the levels of *pre*-*miR156*s expression.

To elucidate whether developmental stage-dependent expressions of miR156 are completely independent from the physical age of their primary shoot apices, levels of miR156 precursors were compared among S1 branches harvested from 4, 5, 6, 7, and 8 weeks old plants. The transcript levels of *pre-miR156a* among S1 branches were declined according to the age of plants. For instance, *pre-miR156a* level in 4WS1, S1 axillary shoot apices from 4-week old Pajares, was 1.9 fold and 4.6 fold higher than 5WS1 and 6WS1 respectively (Fig. 2B). Although the transcript levels of *pre-miR156a* were dramatically reduced from 4WS1 to 6WS1, the levels from 6WS1 to 8WS1 were not reduced further and maintained that quantity, which was similar level observed in 3 weeks old primary shoot apices. Such quantitative results of *pre-miR156a* levels in axillary shoot apices from different ages, the time from germination, and different developmental stages indicate that the molecular behavior of axillary branches is influenced by both their ages when branching started and their developmental stages when harvested. Recent studies concerning floral competency of *A*. *alpina* Pajares reported that all the 5-week old plants but none of 3-week old plants can flower after more than a year of vernalization^19,33^. Thus, it is noteworthy that all the S1 axillary shoot apices from plants with diverse ages show higher levels of *pre-miR156a* than 5-week old primary shoot apices and would not reduce the level below than 3-week old primary shoot apices. It indicates that all the S1 axillary shoots are incompetent to flower regardless of their physical ages after germination.

### After vernalization, some axillary shoot apices expressing high levels of *pre-miR156*s in *A*. *alpina* maintain vegetative phase

**Figure 3:**
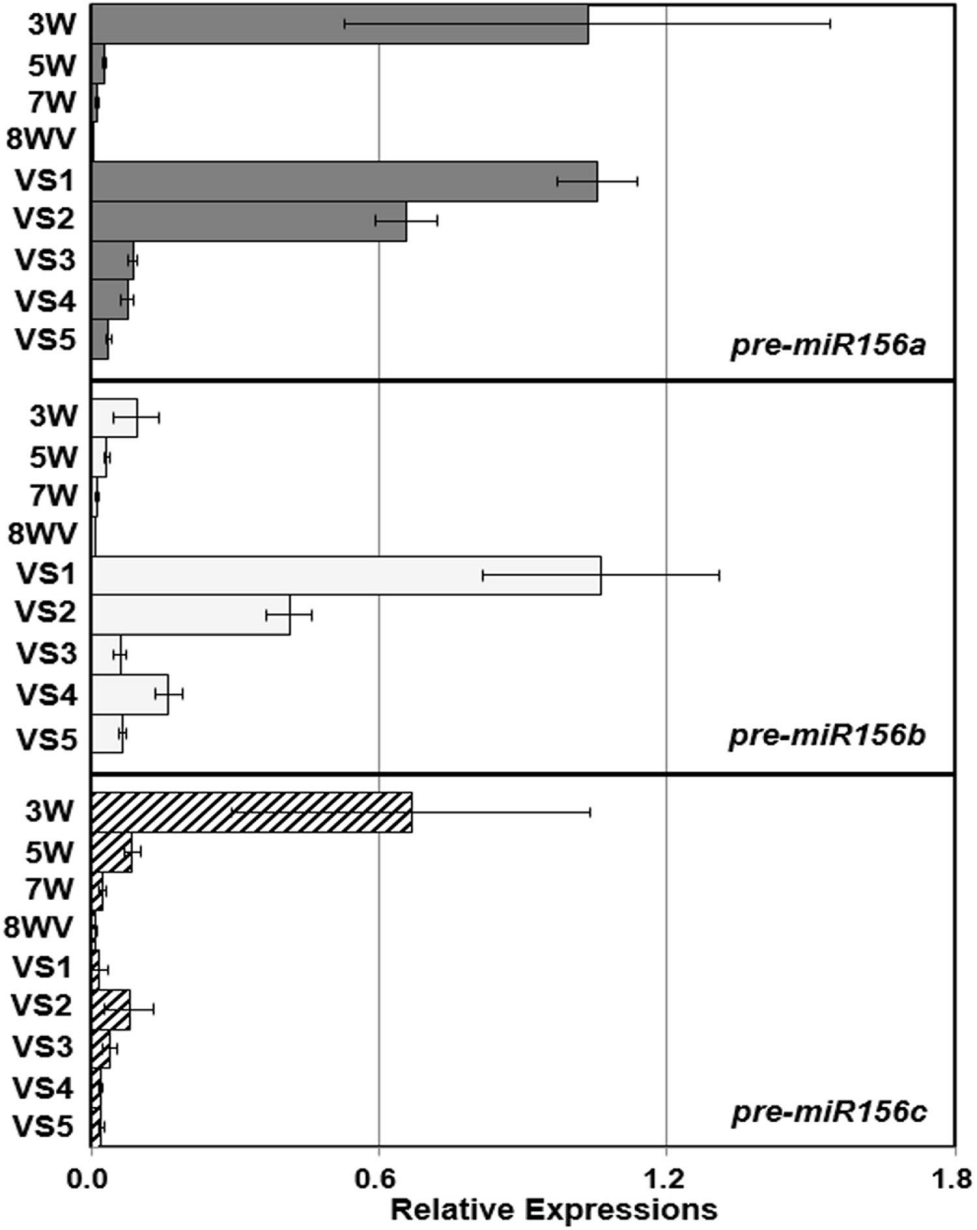
Expression levels of miR156 precursors in the primary and axillary shoot apices of *A*. *alpina* after vernalization. Transcript levels of *pre-miR156a*, *b* and *c* were checked in the plants treated with 12 weeks of vernalizartion after 8-week growth in long days. 8WV, plants vernalized with 12 weeks of cold after 8 weeks of growth in room temperature. VS1 to VS5 indicate S1 to S5 stages of axillary shoot apices from 8WV plants. Primary shoot apices of 3W, 5W, 7W old vegetative plants were compared with axillary branches from 8-week vernalized plants for comparison of miR156 levels.

To make it clear whether miR156 expression levels of primary and axillary shoot apices are related to floral transition, we investigated the transcript levels of *pre-miR156a*, *b* and *c* before and after vernalization. For vernalization treatment, 8-week old vegetative Pajares plants were exposed to 12 weeks of cold (8WV). After vernalization, each axillary branch from these 8WV plants was categorized into VS1 to VS5 according to developmental stages (described in Table S1 and Figure S2C~G). To compare the transcript levels of *pre-miR156s*, we collectively harvested the axillary shoots undergoing the same developmental stage from all node positions. The VS1 and VS2 branches, which were mostly generated during vernalization period, expressed prominently high levels of *pre-miR156a* and *b*. Such levels of *pre-miR156a* and *b* were higher than the levels detected from the primary shoot apices in juvenile phase, i.e, younger than 3 weeks old primary shoots (Fig. 3). As developmental stages were progressed from VS1 to VS5, the transcript levels of *pre-miR156a* and *b* were steadily reduced similar to the pattern observed in the primary shoots. However, the *pre-miR156c* level was very low in the axillary shoot apices after vernalization (Fig. 3).

**Figure 4:**
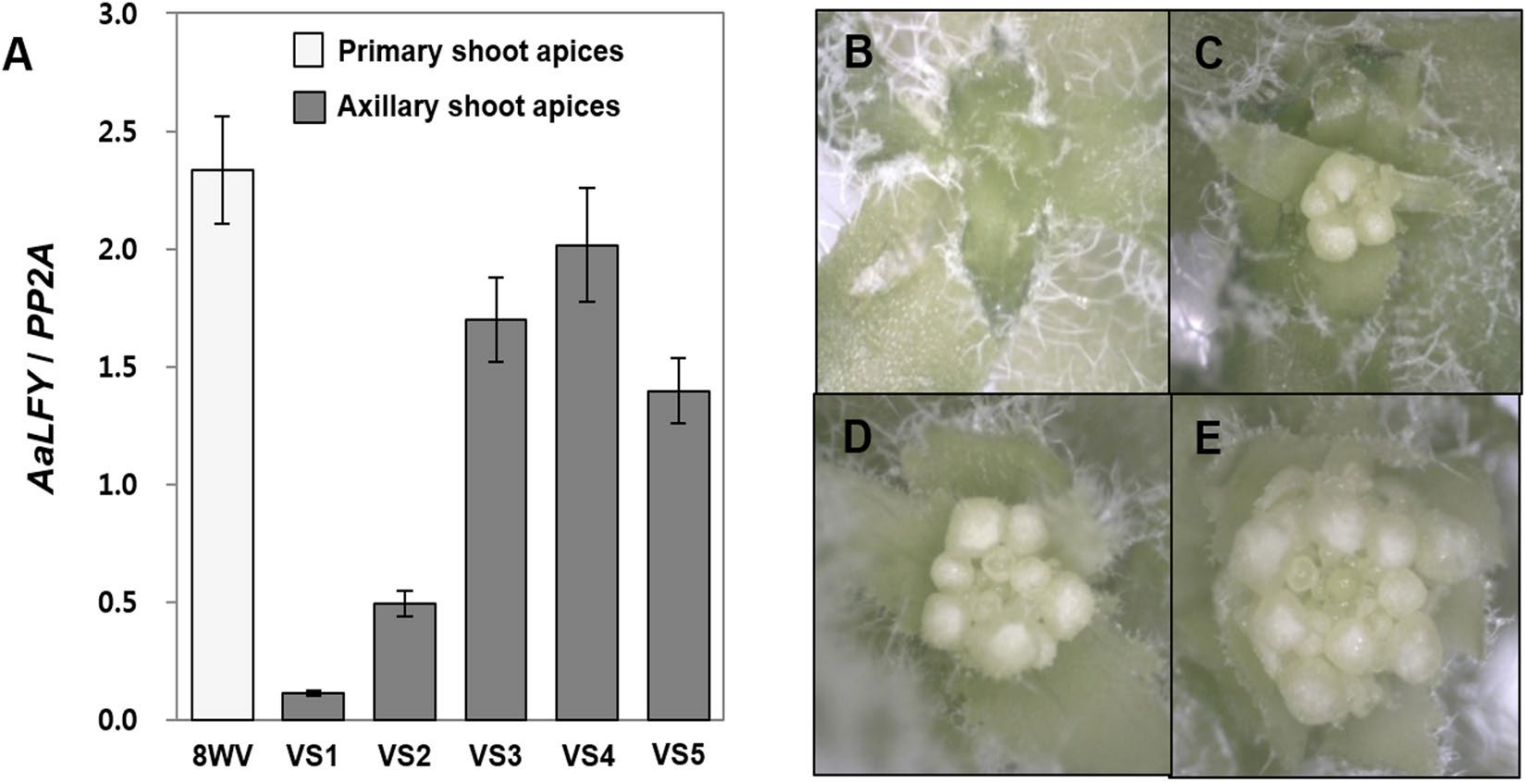
Flowering competence of axillary shoot apices after vernalization in *A*. *alpina* depends on developmental stages. (**A**) Expression levels of *AaLFY* in the primary and axillary shoot apices undergoing various developmental stages after vernalization (8WV, vernalization-treated plants after 8 weeks growth in long days; VS1 to VS5, axillary branches of S1 to S5 stages from 8WV plants). (**B**)~(**E**) Morphologies of shoot apices in different developmental stages. (**B**,**C**) VS2 axillary branches show either vegetative (**B**) or reproductive (**C**) development. (**D**) All of VS3 axillary shoot apices and (**E**) all the primary shoot apices of 8WV plants show inflorescence development.

We also quantified the transcript levels of *LFY* to determine the meristem identity of these axillary branches (Fig. 4A). In contrast to the *pre-miR156*s expressions, *LFY* was eminently expressed in the VS3 to VS5 axillary shoot apices but was low in the VS1 and VS2. Consistent with this, the VS3 to VS5 branches developed floral meristems (Fig. 4D and E). Meanwhile, most of the VS2 and all the VS1 apices were in the vegetative phase even after vernalization (Fig. 4B and C). Our results indicate that some axillary branches, expressing high levels of *pre-miR156*s, maintain vegetative phase even after vernalization.

### miRNA156 levels in all the axillary shoot apices of *A*. *thaliana* Sy-0 are similar independent of developmental stages

To see if asynchronous expression of *miR156* precursors in the axillary shoots of *A*. *alpina* is a unique feature of perennial plant, we compared the expression with that in a close annual relative, *A*. *thaliana*. In Pajares, *pre-miR156b*, *c*, and *d* showed relatively weak expression compared to *pre-miR156a*, thus we focused on *pre-miR156a* level in the axillary shoots of winter annual Arabidopsis ecotype, Sy-0. In contrast to rapid cycling accessions of Arabidopsis such as Columbia (Col) and Landsberg *erecta* (L*er*), Sy-0 shows acropetal development of axillary shoots subtended by cauline leaves. This is quite dissimilar with Col, L*er*, and late-flowering mutants obtained from these genetic background such that they show basipetal development of axillary shoots after flowering^35–38^. Because Sy-0 shows acropetal development of axillary shoots and produces aerial rosette leaves after bolting, the axillary shoots from Sy-0 seem to be equivalent to the axillary shoots developed in Pajares after vernalization.

**Figure 5:**
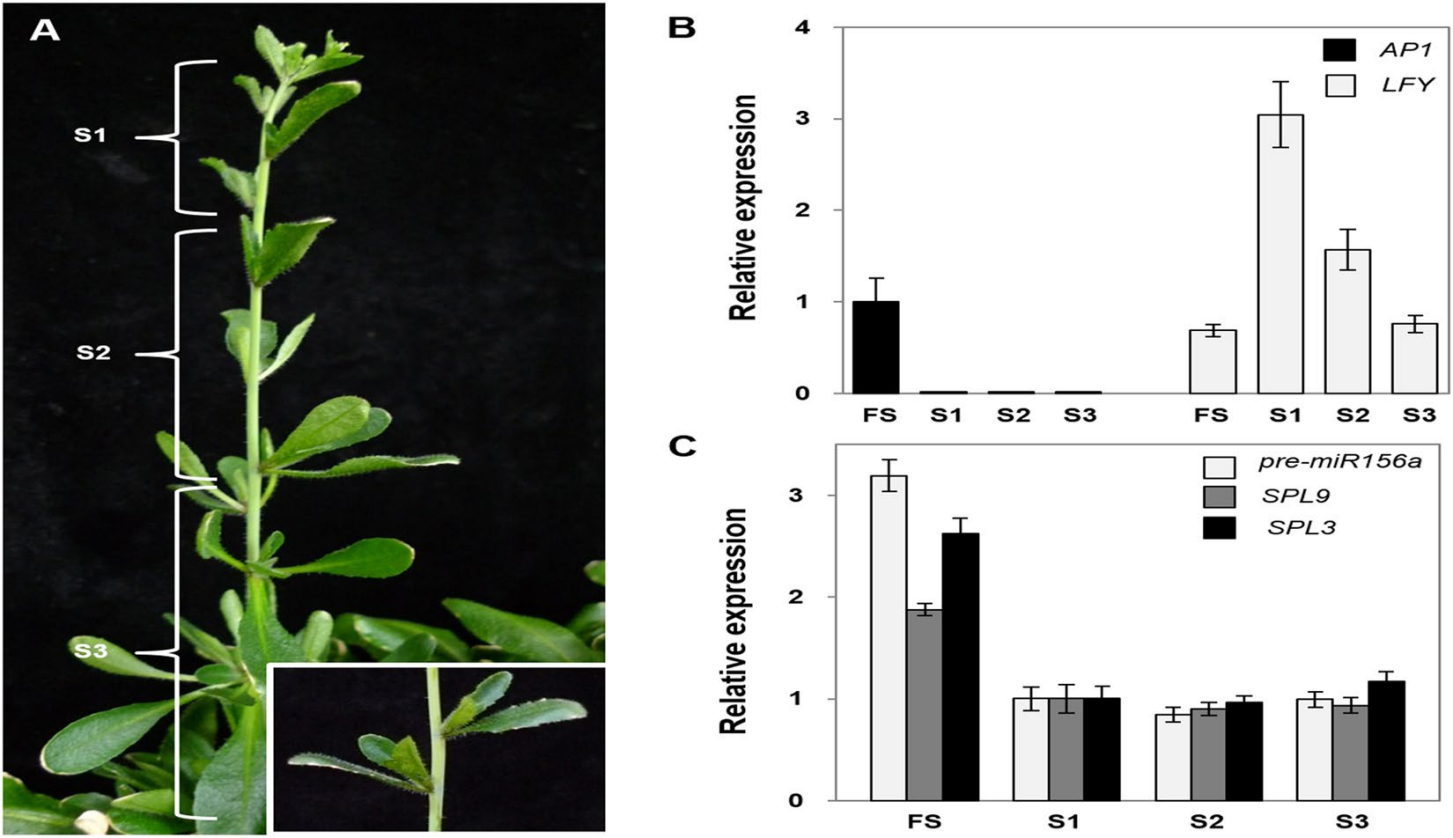
Expressions of several genes in the axillary shoots of winter annual Arabidopsis, Sy-0 were synchronized. (**A**) A 14-week old *A*. *thaliana* Sy-0 grown under long-day condition developed aerial rosettes in diverse developmental stages in axillary branches. Magnified aerial rosette is shown in box. (**B**) Relative expression levels of floral marker genes in the primary shoot apices (FS) and axillary shoot apices (S1, S2 and S3) from 14-week old *A*. *thaliana* Sy-0. (**C**) Relative expression levels of *pre-miR156a*, *SPL3* and *SPL9* in the primary shoot apices (FS) and axillary shoot apices (S1, S2, and S3) from 14-week old *A*. *thaliana* Sy-0.

The axillary branches of Sy-0 can transit to reproductive phase after producing more than ~12 aerial rosette leaves. These branches containing aerial rosettes of 14-week old Sy-0 were classified into 3 categories based on the number of aerial rosette leaves and the position of axillary branching (the description of each categories are in Fig. 5A and Table S2). The S1 axillary branches have less than 4 aerial rosette leaves and developed more lately than the other axillary branches. To check whether all the axillary branches are at the vegetative phase, expressions of *APETALA1* (*AP1*) and *LEAFY* (*LFY*), two floral meristem identity genes, were analyzed in S1~S3 shoot apices. Floral organ specific *AP1* was expressed only in the flowering primary shoot apices, but not detected in their axillary shoot apices. On the other hand, S1~S3 axillary shoot apices expressed *LFY* higher than the primary shoot apices. As axillary branches developed from S1 to S3, *LFY* expression was decreased. Since *LFY* is expressed not only in floral organs but also in leaf primordia^39^, it is likely that S1 containing more primordia than S2 or S3 exhibits higher level of *LFY* (Fig. 5B). The transcript level of *pre-miR156a* was examined in these vegetative S1~S3 and primary shoot apices from 14-week old Sy-0. All stages of axillary shoot apices showed similar levels of *pre-miR156a*. The other miR156 precursors were expressed too low to compare their values (Figure S6). The transcript levels of *SPL3* and *SPL9*, direct target genes of miR156, were also expressed in similar level in all the axillary shoot apices (Fig. 5C). These results indicate that the level of miR156 expression in axillary shoot apices is irrelevant to the developmental stages of axillary branches in the annual *A*. *thaliana* Sy-0. Therefore, our results demonstrate that the developmental fate of all the axillary branches from the same Sy-0 plant is synchronized at a molecular level.

### Differential responses to vernalization in *A*. *alpina* Pajares and *A*. *thaliana* Sy-0

**Figure 6:**
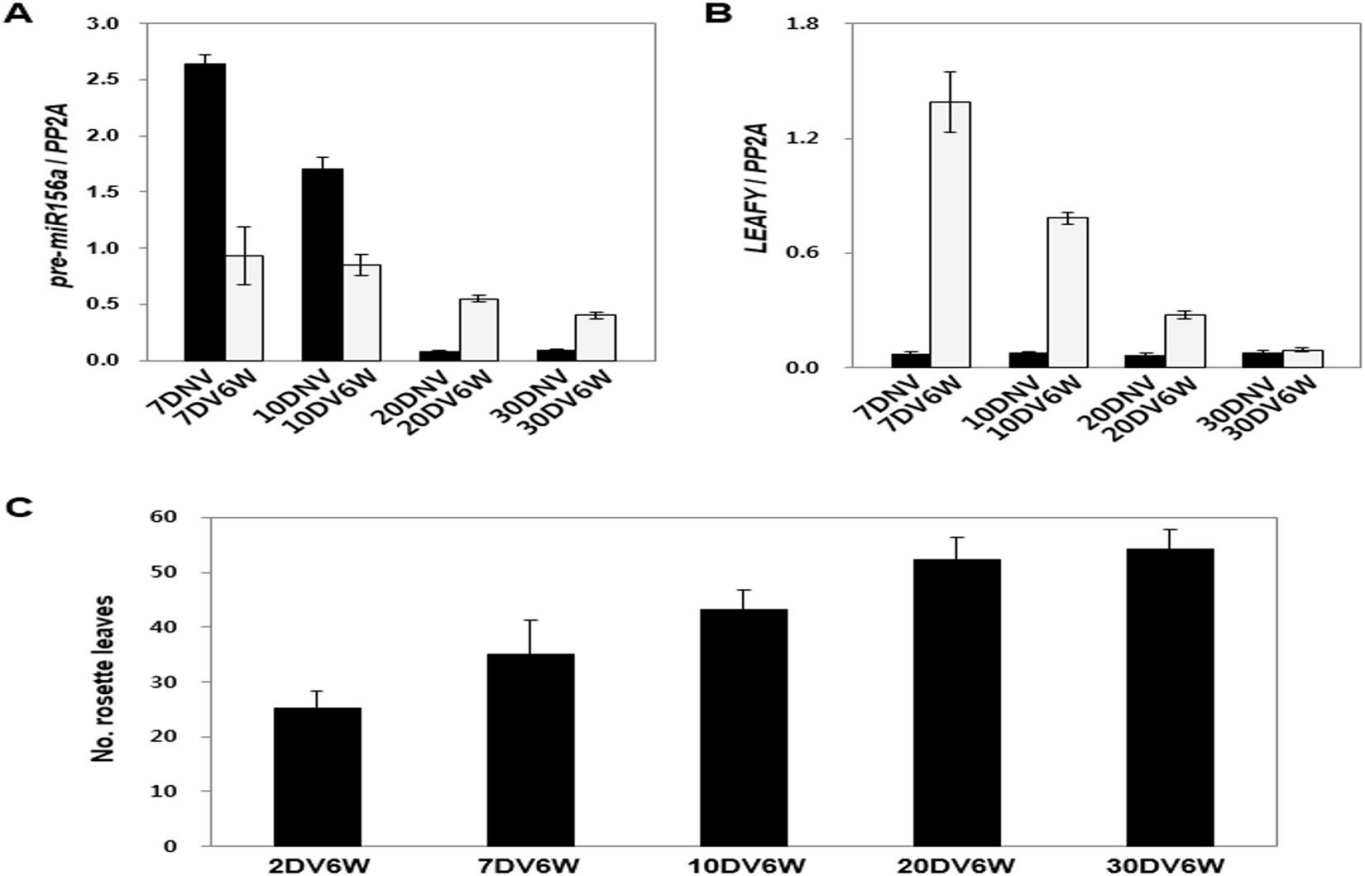
Vernalization response of *A*. *thaliana* Sy-0 according to age. (**A**) Expression levels of *pre-miR156a* in the primary shoot apices of 7, 10, 20, and 30-day-old Sy-0 before vernalization (black bars) and after 6 weeks vernalization (white bars) treatment. (**B**) Expression levels of *LFY* were analyzed before (black bars) and after (white bars) vernalization in Sy-0. Plants in variable ages from 7 days to 30 days old were exposed to 6 weeks vernalization. (**C**) Flowering time was measured by the number of rosette leaves produced when flowering. Plants in variable ages from 2 days to 30 days old were exposed to 6 weeks vernalziation.

In Pajares, 5-week old plants can transit from vegetative to reproductive phase if they are sufficiently exposed to vernalization treatment, but 3-week old plants fail to progress reproductive phase even though they are exposed to prolonged cold environment^33^. Expression levels of miR156 in juvenile Pajares plants, younger than 3-week old, are almost unchanged during vernalization, whereas the levels drop rapidly after returning to warm environment^19^. Consistently, our experiments also showed that all the 5-week old Pajares plants perfectly respond to vernalization but all the 3-week old plants failed to flower even after 12 weeks vernalization (n = 64, respectively). However, in case of 4-week old Pajares, 31.75% of vernalized plants among 63 flowered. To decide whether juvenility is also an important factor for vernalization response in winter annuals of Arabidopsis, we checked vernalization effect according to the expression levels of *pre-miR156a* and the ages of Sy-0. Transcript levels of *pre-miR156a* from the 7-day old Sy-0 was dramatically reduced to 35.0% after 6 weeks of vernalization, as opposed to juvenile Pajares which maintains high levels of *pre-miR156a* during vernalization^19^. In case of 20-day and 30-day old Sy-0, the transcript levels of *pre-miR156a* increased to 7-fold and 4.3-fold higher after vernalization compared to plants before vernalization, instead of decreasing. Therefore, the transcript levels of *pre-miR156a* at the end of 6-week vernalization were considerably different in each age (Fig. 6A). We also checked the transcript levels of *LFY* for these Sy-0 plants vernalized at different ages. Before vernalization, the transcript levels of *LFY* were very low since Sy-0 is a very late-flowering winter annual. However, after vernalization, *LFY* expression in Sy-0 was highly activated and the activation of *LFY* was stronger in juvenile plants than older plants (Fig. 6B). In contrast to Sy-0, *AaLFY* in juvenile Pajares was not activated by vernalization. *AaLFY* expression was highly activated by vernalization only in floral-competent 5-week old Pajares; *AaLFY e*xpression was increased to about 7-fold at the end of vernalization comparing to non-vernalized condition (Figure S7). Then, vernalization response of Sy-0 according to physical ages after germination was analyzed. Sy-0 showed earlier flowering because of stronger vernalization response, if exposed to vernalization in younger stage (Fig. 6C). This result shows that Arabidopsis winter annuals do not have juvenile insensitivity to vernalization, instead juvenile Sy-0 is more sensitive to vernalization than adult plants. Therefore, developmental maturity is a critical factor for vernalization-mediated flowering in perennial Pajares whereas winter annual Sy-0 can respond to vernalization regardless of the ages when vernalized.

## Discussion

In terms of longevity, plants can be largely divided into perennial and annual plants^2^. Most of perennial plants are polycarpic, that is, repetitively producing flowers every year whereas annual plants are monocarpic, producing flowers once in a life time. Recent studies using perennial plants *Arabis alpina* and *Cardamine flexuosa* provided important insights of a molecular nature of perenniality but still we are devoid of complete understanding^17,19^. To provide molecular basis of perenniality, we directly compared the molecular nature of *A*. *alpina* Pajares and *Arabidopsis thaliana* Sy-0. In both Arabidopsis winter annual Sy-0 and perennial *Arabis alpina* Pajares, axillary branches in various developmental stages are produced in the same plant (Figs 1 and 5A, Tables S1 and S2). However, in Pajares, each axillary branch expresses differential levels of *pre-miR156*s according to its developmental stage and age of a primary shoot when branching initiated (Fig. 2). Such differential expression of miR156 precursors depending on the developmental stages of axillary apices was observed even after primary shoots were in flowering phase (Fig. 3). In contrast to this, *pre-miR156a* levels in the axillary branches from the same Sy-0 were similar irrespective of developmental stages, thus synchronized (Fig. 5C). Since miR156 is a general key player in phase transitions of plants and *MIR156B*-overexpressing Pajares fails to flower even after long-term cold exposure^19^, such asynchronous expression of miR156 in the axillary branches of Pajares seems to be the basis of maintenance of vegetative shoots after winter. For example, some axillary branches of Pajares expressing high *pre-miR156*s levels maintain vegetative phase even though main shoots are under flowering phase (Figs 3 and 4). On the contrary, Sy-0 shows holistic flowering, because it cannot maintain vegetative growth due to low levels of *pre-miR156a* (Fig. 5B and C). Therefore, our results clearly demonstrate that variation of miR156 levels in axillary branches of Pajares confers polycarpic perenniality but synchronous reduction of miR156 levels in the axillary branches of Sy-0 confers monocarpic traits.

The axillary shoots subtended by cauline leaves in Arabidopsis, which is produced after floral transition, may not be comparable to the axillary shoots produced in *A*. *alpina*. Because the axillary shoots produced in *A*. *alpina* are developed acropetally on average, asynchronous though, whereas axillary shoots produced after floral transition in rapid cycling accessions of Arabidopsis are developed basipetally^35–38^. In addition, the molecular basis of axillary shoots developed during vegetative phase, which is produced in the axils of rosette leaves, are dissimilar with the axillary shoots developed during reproductive phase, which is produced in the axils of cauline leaves in Arabidopsis^41^. However, there is a contradictory report showing that axillary shoots produced during reproductive phase in a winter annual Arabidopsis are developed acropetally^40^, which is similar to the axillary shoot development in *A*. *alpina*. Sy-0 is another winter annual accession of Arabidopsis^42,43^ and has a unique feature developing aerial rosettes, which are unusual vegetative leaves at the nodes of elongated stem produced after bolting^37,42–44^. Furthermore, Sy-0 shows acropetal development of axillary shoots in contrast to rapid cycling accessions of Arabidopsis or late-flowering mutants derived from such accessions (Fig. 5A). Therefore, the axillary shoots developed in Sy-0 during reproductive phase are more likely to the axillary shoots produced in Pajares. To confirm such hypothesis, further molecular analyses will be required using molecular markers specific for axillary meristem development.

The heteroblasty caused by differential vegetative phase transitions is more common in perennial plants^46,47^. For instance, morphological and physiological characters, such as plastochron, phyllotaxis, internode length, thorniness, photosynthetic efficiency, adventitious rooting, disease and insect resistance, are distinguishable between juvenile and adult phases^46^. Likewise, *Arabis alpina* Pajares shows typical heteroblastic characteristics such that leaves produced at a juvenile phase are relatively small and simple compared to leaves produced at an adult phase. The basalmost 4 leaves of primary shoots show such juvenile morphology in addition, the shoot apices developed within about 3 weeks after germination expressed *pre-miR156*s relatively high levels, thus are insensitive to vernalization^19^. Similar with primary shoots, basalmost 3 leaves of axillary branches of Pajares are also small and having smooth margins (Figures S1 and S2). The axillary branches produced only 3 leaves are categorized as developmental stage 1 (S1). The S1 axillary shoot apices express high levels of *pre-miR156*s, thus are unable to respond to vernalization. In contrast, Arabidopsis Sy-0 does not undergo such juvenile phase incompetent to vernalization response. Instead, Sy-0 shows higher sensitivity to vernalization at younger stage. Such difference seems to be due to the differential maintenance of *pre-miR156*s levels after vernalization in juvenile stage. In Pajares, the expression levels of *pre-miR156*s are maintained during vernalization^19^, whereas in Arabidopsis Sy-0, miR156 levels are decreased if vernalized at younger ages but increased if vernalized at old ages (Fig. 6A). Therefore, Arabidopsis winter annuals show higher sensitivity to vernalization at younger ages (Fig. 6C). The aim of life in monocarpic annuals is maximizing the number of offsprings by exhausting most of their resources. On the other hand, a life strategy of polycarpic perennials is extension of lifespan as long as possible through multiple times of flowering^2^. In *Arabis alpina* Pajares, vernalization-mediated flowering branches senesce after reproduction like annual plants^48^. Therefore, polycarpic perennials including Pajares require branches in juvenile phase, insensitive to floral inductive signals such as vernalization, to maintain vegetative growth, which allows sustaining perennial traits.

A molecular study in perennial plants is still rare since it has many obstacles, for instance, long generation time, and difficulties in generating transgenic plants. Thus, our study to compare the molecular differences in miR156 expressions and vernalization responses between the perennial *Arabis alpina* and the close relative annual *Arabidopsis thaliana* will be useful for future study. The most urgent question is the molecular mechanism behind the synchronous and asynchronous expression of miR156 in the axillary branches in annuals and perennials.

## Materials and Methods

### Plant materials and growth conditions

*A*. *alpina* Pajares and *A*. *thaliana* ecotype Sy-0 seeds were surface sterilized in 75% ethanol and 0.05% tween-20 solution. After sterilization, seeds were sown on one-half-strength MS medium supplemented with 1% (w/v) sucrose and 1% (w/v) plant agar. The Pajares and Sy-0 seeds were stratified under dark at 4 °C for 10 days and 3 days, respectively. After stratification, the seeds were germinated on MS medium, then seedlings were transplanted to soil under controlled condition of 16-hour light and 8-hour dark at 22 °C. For long-term cold treatment, vernalization, plants were transferred to vernalization chamber (8-hour light and 16-hour dark at 4 °C).

### Characterization of miR156 precursors in *Arabis alpina* Pajares

To obtain the information of nucleotide sequences of miR156 precursors in *Arabis alpina* Pajares, BLAST tool of NCBI (ftp://ftp.ncbi.nlm.nih.gov/blast/executables/blast+/) was used with Genome database of Pajares^34^ (https://www.ncbi.nlm.nih.gov/bioproject/PRJNA241291). Based on the genomic sequences, we cloned six homologs of miR156 precursors which are highly similar with miR156 precursors in Arabidopsis. The sequences covering target-binding site were used for quantitative RT-PCR with the specific primers presented in Table S3. Nucleotide sequences of miR156 precursors in *A*. *alpina* Pajares are annotated (Table S4).

### Sampling of axillary shoot apices in *Arabis alpina* Pajares and *Arabidopsis thaliana* Sy-0

For expression analysis of miR156 precursors in various stages of axillary branches of *Arabis alpina* Pajares, S1 to S5 stages of axillary shoot apices were harvested from 8-week old Pajares before and after vernalization (Figs 2 and 3). The axillary shoot apices undergoing the same developmental stages were collectively harvested regardless of the node positions.

### Microscopic Analysis

To determine meristem identity, microscopic analysis was performed in various stages of axillary branches of Pajares after long-term cold treatment. Vegetative 8 week old Pajares, which have S1~S5 axillary branches, were exposed to vernalization for 12 weeks. The shoot apices of primary and axillary branches were observed by digital light-microscope (DIMIS-M^®^, CMOS sensor) before returning to warm temperature.

### Transcript expression analysis

For gene expression analysis, total RNA was extracted from apical and axillary shoot apices in certain developmental stages and chronological ages using the RNeasy^®^ plant mini kit (QIAGEN 74904). Contaminated genomic DNA was eliminated with recombinant DNaseI (Takara 2270A). cDNA was synthesized using total RNA with reverse transcriptase (Fermentas EP0441) and oligo(dT). Quantitative PCR was performed using the iQ^TM^ SYBR^®^ Green Supermix (Bio-Rad 170-8882) and analyzed by the CFX96 real-time PCR detection system.

### Oligonucleotide Primers

The sequences of oligonucleotide primers used in this work were listed in supplemental Table S3.

## Electronic supplementary material

Supplementary Information
