# Author Correction: Comparative analysis of molecular and physiological traits between perennial *Arabis alpina* Pajares and annual *Arabidopsis thaliana* Sy-0

**DOI:** 10.1038/s41598-018-21281-5

**Published:** 2018-03-01

**Authors:** Jong-Yoon Park, Hoyeun Kim, Ilha Lee

**Affiliations:** Laboratory of Plant Developmental Genetics, School of Biological Sciences, Plant Genomics & Breeding Institute, Seoul National University, Seoul, 08826 Korea

Correction to: *Scientific Reports* 10.1038/s41598-017-13606-7, published online 17 October 2017

The Acknowledgements section in this Article is incomplete.

“*Arabidopsis thaliana* ecotype Sy-0 seeds were supplied by Vojislava Grbic (Department of Biology, The University of Western Ontario, 1151 Richmond Street, London, Ontario, Canada N6A5B7).”

should read:

“*Arabidopsis thaliana* ecotype Sy-0 seeds were supplied by Vojislava Grbic (Department of Biology, The University of Western Ontario, 1151 Richmond Street, London, Ontario, Canada N6A5B7). This work was supported by National Research Foundation of Korea (NRF) Grants from the Korean Government, No. 2014023132 and NRF-2017R1D1A1B03030489.”

