# Supplementary material for: Comparative analysis of molecular and physiological traits between perennial Arabis alpina Pajares and annual Arabidopsis thaliana Sy-0
Source: Sci Rep. 2017 Oct 17;7:13348. doi: 10.1038/s41598-017-13606-7 (PMC5645391; doi:10.1038/s41598-017-13606-7)
Supplement: Supplementary file 1 — Supplementary Information [file 41598_2017_13606_MOESM1_ESM.pdf]

# Supplementary Information

# Comparative analysis of molecular and physiological traits between perennial *Arabis alpina* Pajares and annual *Arabidopsis thaliana* Sy-0.

Jong-Yoon Park, Hoyeun Kim and Ilha Lee*

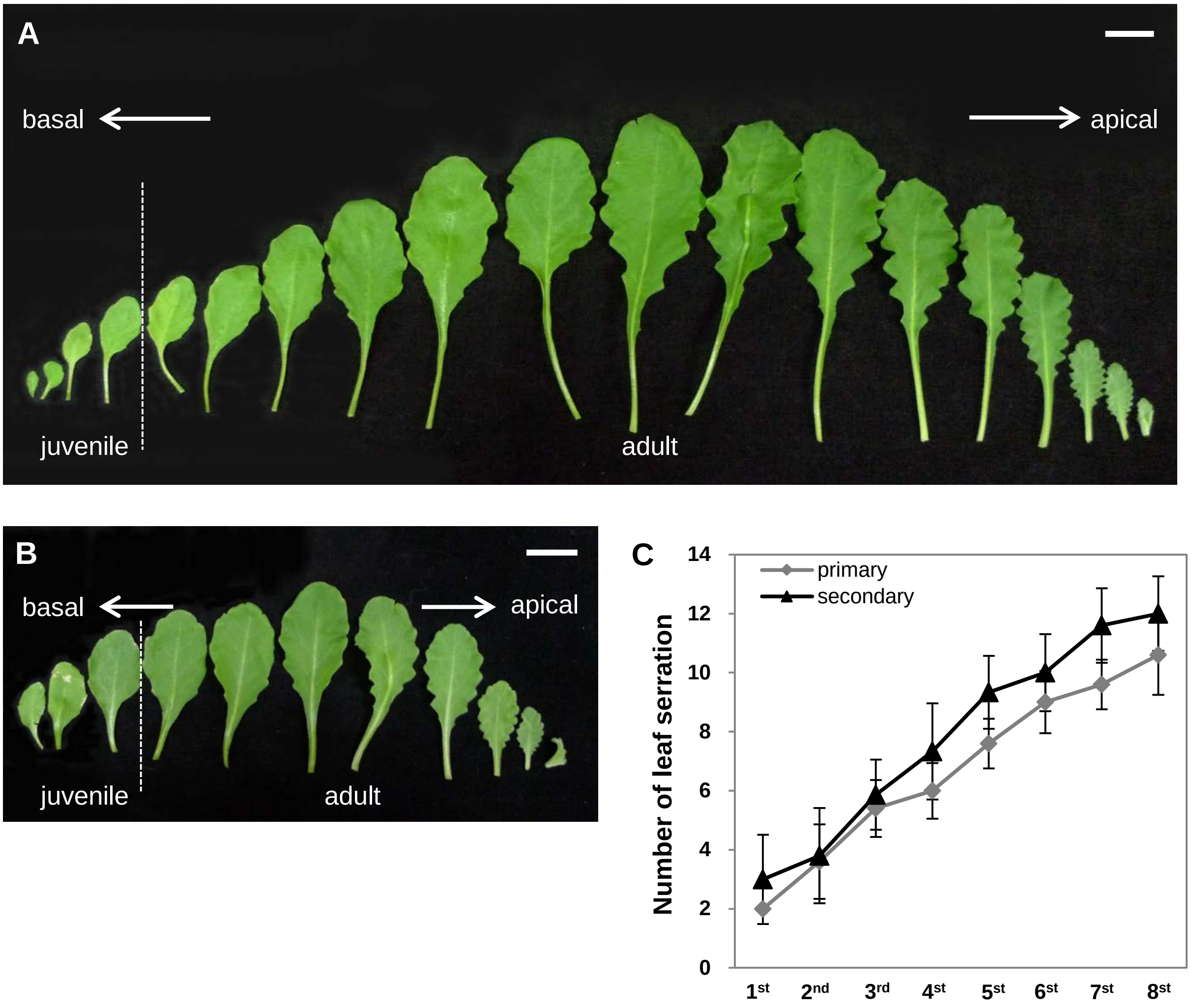


**Figure S1. Leaf developments of both primary and axillary shoots of *A. alpina* Pajares show similar transition from juvenile to adult phase.**

(A) Morphology of the leaves from a primary shoot from 9-week old *A. alpina* Pajares showing both juvenile and adult leaves. Scale bar=1cm

(B) Morphology of the leaves from an axillary branch generated at the 2$^{nd}$ node showing both juvenile and adult leaves. Scale bar = 1cm

(C) Average numbers of leaf serration from the 1$^{st}$ to the 8$^{th}$ leaves developed after cotyledon (n=15). The number of leaf serration increases according to developmental phase, which is observed in both primary and axillary shoots.

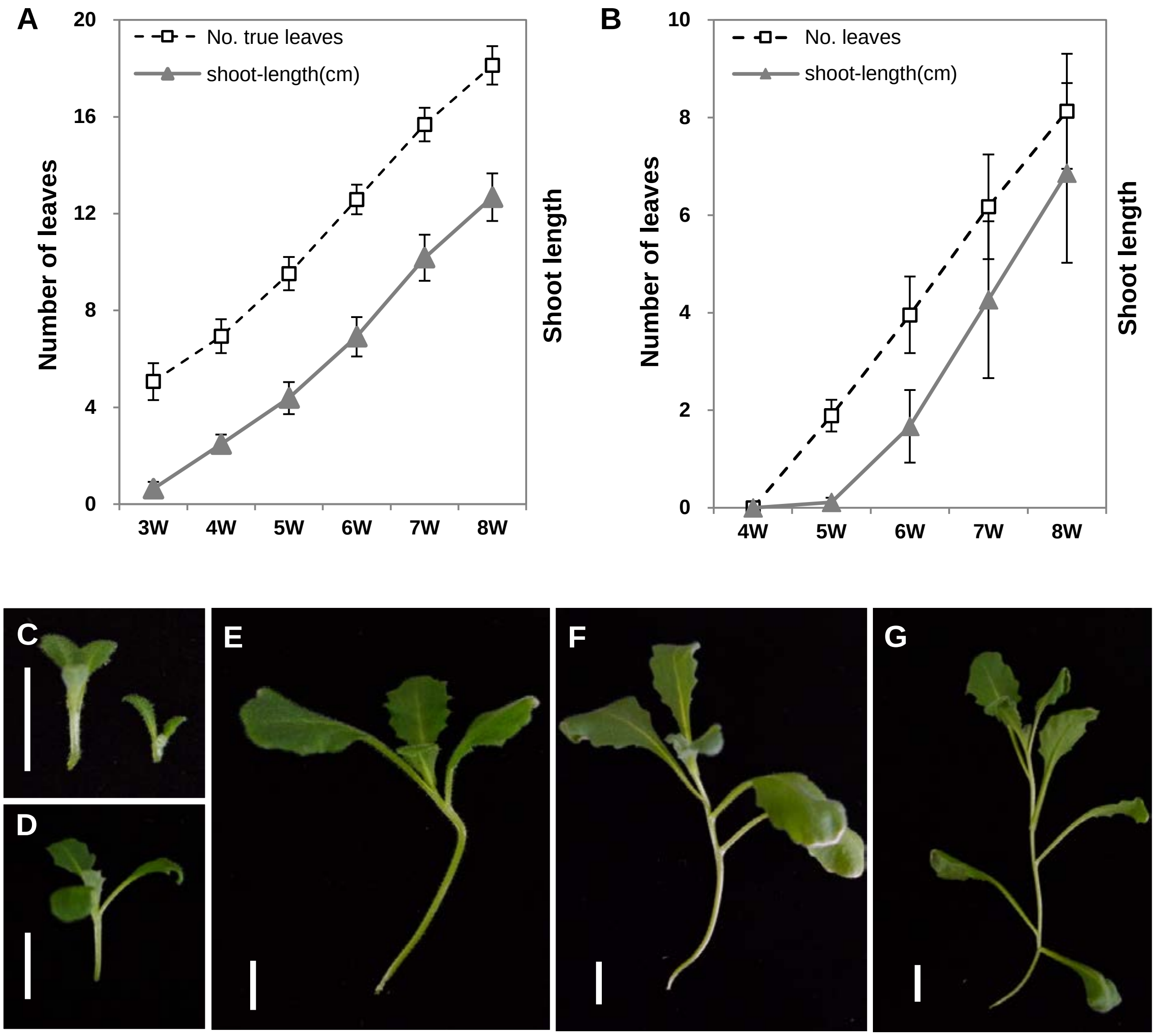


**Figure S2. Developmental patterns of primary and axillary shoots in *A. alpina* Pajares and categorization of developmental stages from S1 to S5.**

(A) The number of true leaves and  the shoot length from primary shoots were measured weekly from 3 weeks to 8 weeks. (n=24).

(B) The numbers of true leaves and the shoot length from axillary shoots were measured, weekly. The axillary branches produced at the 2$^{nd}$ node from cotyledons were used for this measurement. (n=24)

(C~G) Various developmental stages of axillary branches were categorized as S1~S5 stages of which characterizations were described in Table S1. (C) to (G) are representative photos for stage S1 to S5. (Scale bar=1cm)

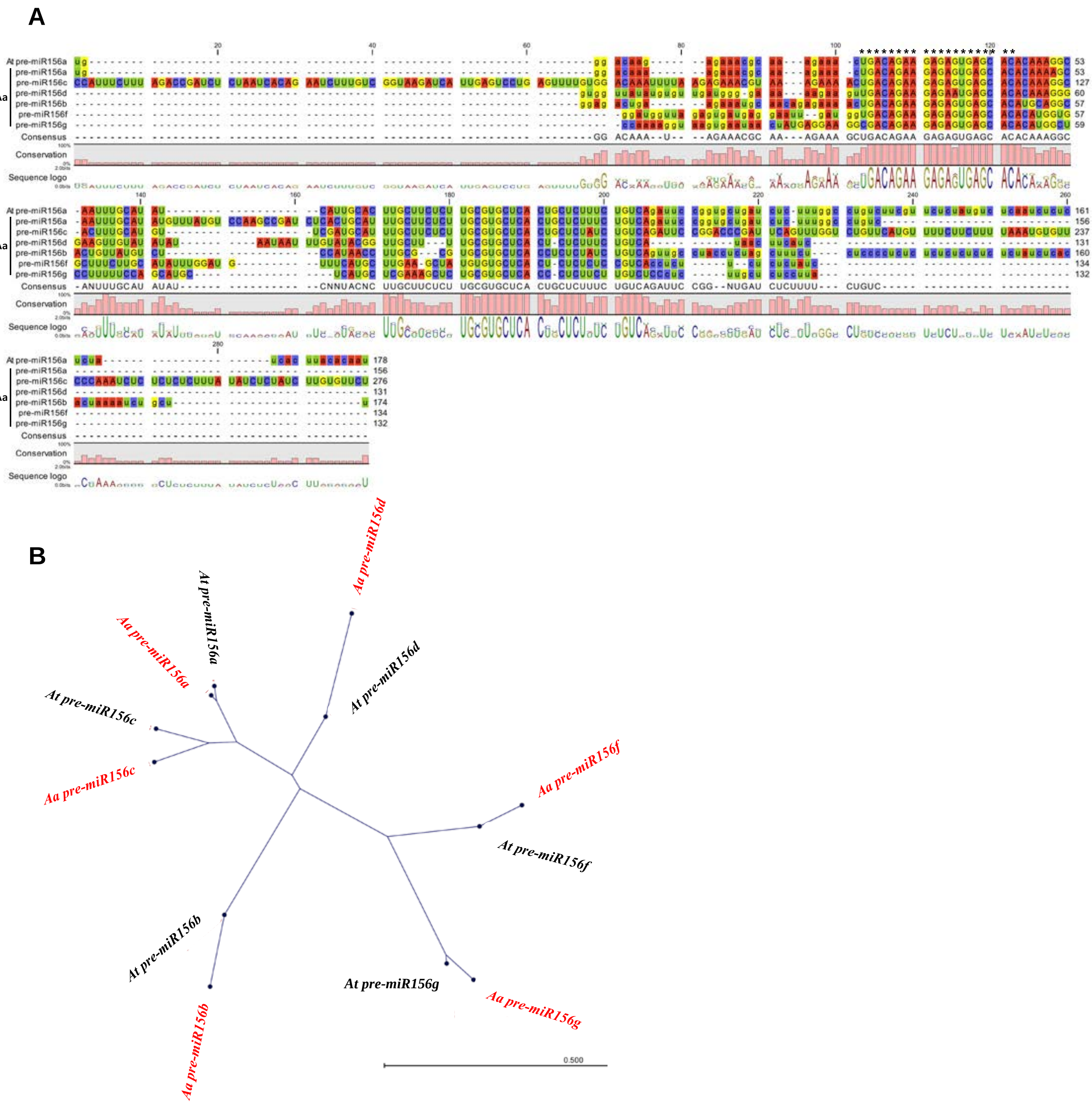


**Figure S3. Six homologs of miR156 precursors in *Arabis alpina* Pajares.**

(A) Multiple sequence alignment of nucleotide sequences of miR156 precursors from Arabidopsis and *Arabis alpina* Pajares was obtained by using CLC Main Workbench version 7.7.1. Conservation ratios are indicated as bar graph. Target binding sites of miR156s are highly conserved in both *A. alpina* and Arabidopsis (asterisks).

(B) A phylogenetic tree of miR156 family from Arabidopsis and *Arabis alpina* Pajares. Evolutionary analyses were conducted in CLC Main Workbench version 7.7.1.

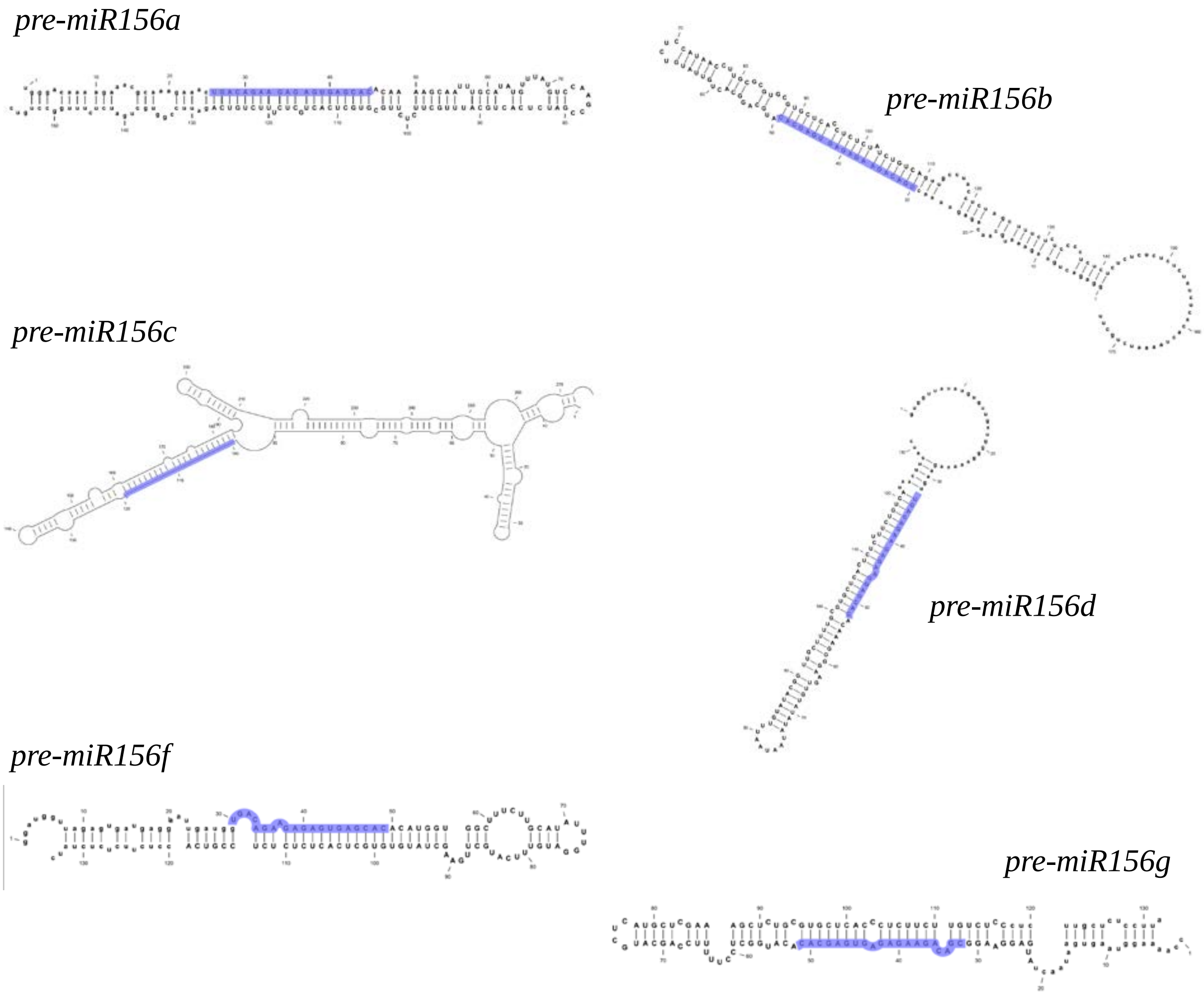


**Figure S4. Secondary structure prediction of six homologs of miR156 precursors in *Arabis alpina* Pajares.**

Transcripts of six miR156 precursors containing the target binding site were cloned. Based on these nucleotide sequences, secondary structures of miRNAs were predicted by RNA structure tool of CLC Main Benchwork version 7.7.1. Conserved target binding sequences are highlighted with purple color.

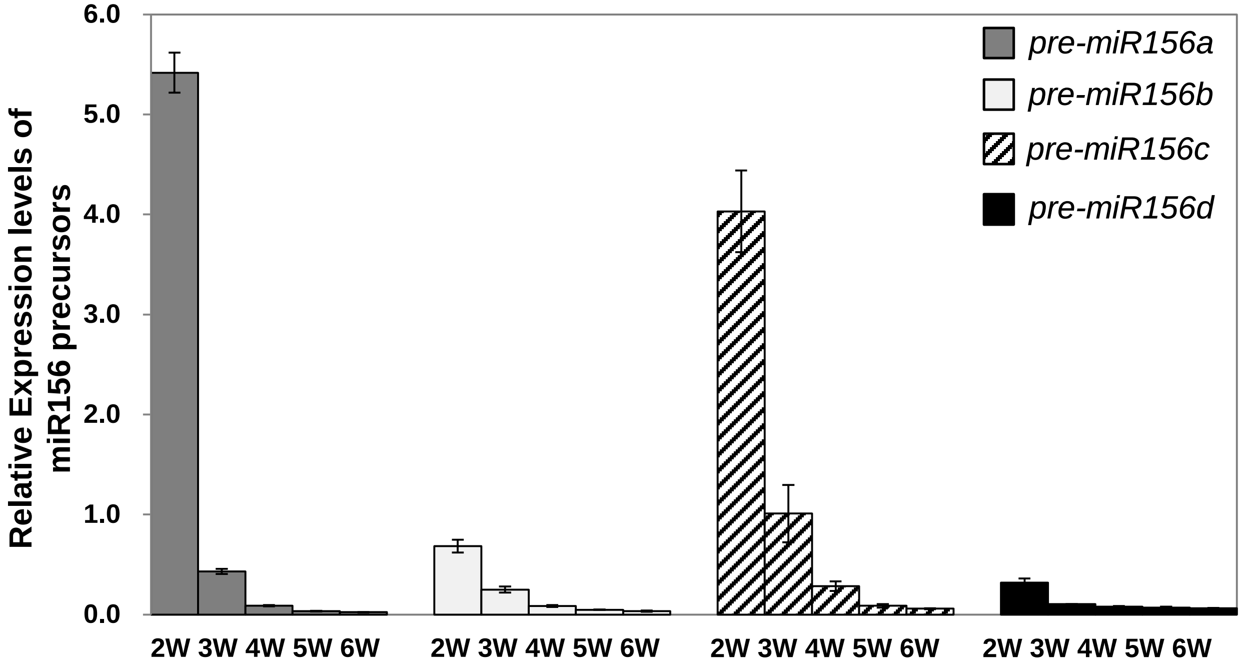


**Figure S5. Relative expression levels of miR156 precursors in the primary shoot apices of *A. alpina* during 2 to 6-week vegetative growth.** Quantitative RT-PCR results show that the transcript levels of *miR156a*, *b*, *c* and *d* precursors are declined age-dependently in the primary shoot apices during vegetative growth.

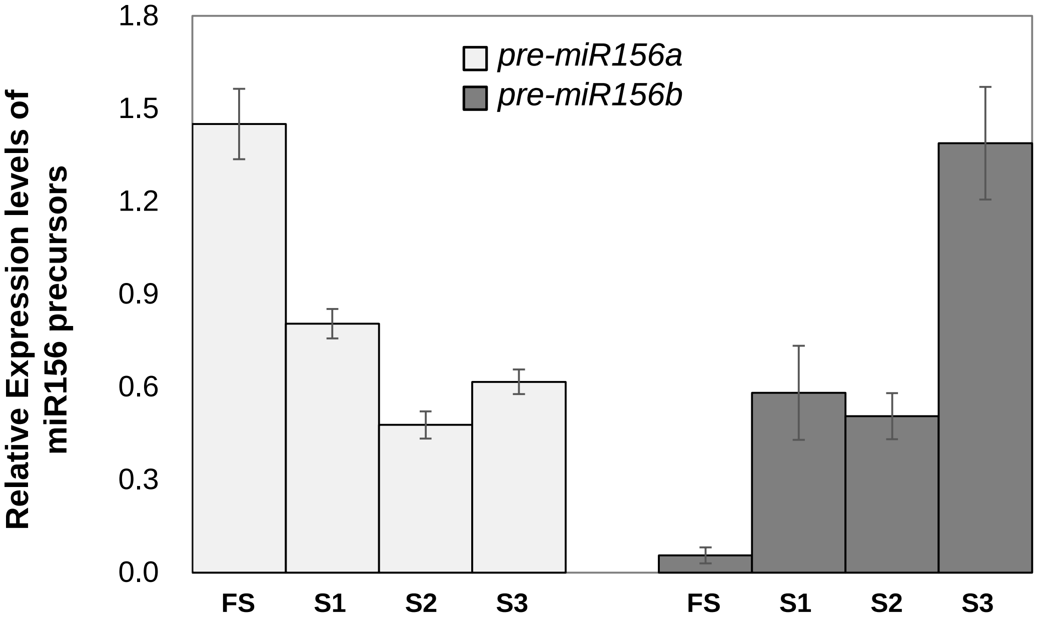


**Figure S6. Relative expression levels of miR156 precursors in *Arabidopsis thaliana* Sy-0.**

Relative expressions of *pre-miR156a* and *b* in primary floral shoot apices (FS) and axillary shoot apices (S1, S2 and S3) from 14-week-old Sy-0. Expressions of other precursor genes such *as pre-miR156c*, *d*, *e*, *f* and *h* were too weak to analyze.

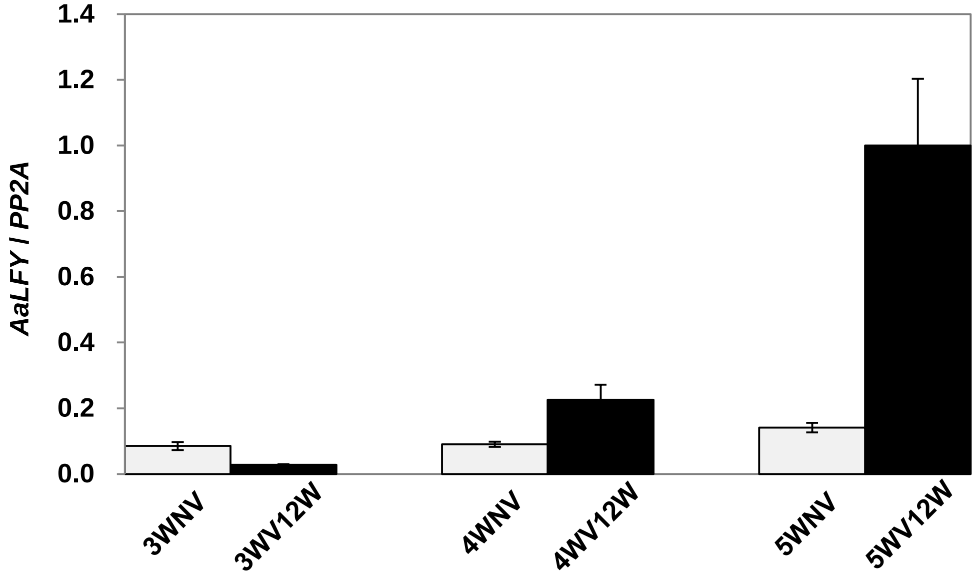


**Figure S7. Vernalization mediated floral transition in the primary shoot apices of Pajares at different developmental ages.**

Comparison of the transcript levels of *AaLFY* before (white bars) and after (black bars) 12 weeks of vernalization at different ages.

| Number of elongated internodes | Number of leaves | Developmental stage |
|---|---|---|
| 0~1<br>(branch length≤0.5cm) | ≤3 | Stage 1 (S1) |
| 1<br>(branch length>0.5cm) | ≤4 | Stage 2 (S2) |
| 2~3 | ≤6 | Stage 3 (S3) |
| 4~5 | ≤8 | Stage 4 (S4) |
| more than 6 | ≥8 | Stage 5 (S5) |

**Table S1. A categorization of developmental stages of axillary shoots, S1 to S5, according to the numbers of elongated internodes and leaves generated from axillary branches.**

| Number of aerial rosette leaves | Node position of shoot branching | Developmental stage |
|---|---|---|
| 3~4 | $1^{st}$~$3^{rd}$ nodes from primary shoot apex | stage 1 (S1) |
| 5~8 | $4^{th}$~$7^{th}$ nodes from primary shoot apex | stage 2 (S2) |
| 9≤ | after $8^{th}$ nodes from primary shoot apex | stage 3 (S3) |
| · | primary shoot apex | Floral stage (FS) |

**Table S2. Classification of developmental stages of axillary branches in *A.thaliana* Sy-0.** Developmental stages of axillary branches from 14 weeks old winter annual, Sy-0 were categorized according to the number of aerial rosette leaves and the position of branching.

| Species | Gene | Sense | Primer Sequences (5'-3') | Reference |
|---|---|---|---|---|
| *A. alpina* | pre-miR156a | F | TGGGACAAAAGAAACGCAAAG | Bergonzi *et al*., 2013 |
| | | R | GACAGGCCAAAGAGATCAGC | |
| | pre-miR156b | F | GAGACTGAAGAAATGCAACAGAG | |
| | | R | AAGCAGATTTTAGTGTGAGATAGAG | |
| | pre-miR156c | F | CCATTTCTTTAGACCGATCTCTAA | |
| | | R | AGAACACAAGATAGAGATATAAAGAG | |
| | pre-miR156d | F | GTGGTTATATGTGTTGATGGGGA | |
| | | R | GATGAAGTTATGACAGAAAGAGAGT | |
| | pre-miR156f | F | GGATGGTTAGAGTGATGAGGAAT | |
| | | R | GATAGAGAGAAGAGGTGACGG | |
| | pre-miR156g | F | CCAAAAGGTAAGTGAATAACTATGAG | |
| | | R | TAAGGAGAGCAAGAGGGAGACA | |
| | *SPL9* | F | GTTGTCCTAATGATATGTCTCCTG | |
| | | R | GTCATAAGCCCTTGTGTTCTCAC | |
| | *LFY* | F | CGAACCAAGTGTTTAGGTACGC | |
| | | R | CGTTCTCGCCGCGTTCTTTAAA | |
| | *PP2A* | F | AGTATCGCTTCTCGCTCCAG | Bergonzi *et al*., 2013 |
| | | R | AACCGGTTGGTCGACTATTG | |
| *A. thaliana* | pre-miR156a | F | GGACAAGAGAAACGCAAAGAAAC | |
| | | R | AGTGAGCACGCAAGAGAAGCAAG | |
| | pre-miR156b | F | AGCACATGCAGGCACTGTTAT | |
| | | R | TCAAGCAGGCAGAGATAGGC | |
| | *SPL3* | F | TGAGTTTGATGAAGCCAAGC | |
| | | R | TCACACGTTTCACCGTCTTT | |
| | *SPL9* | F | AGAACATTGGATACAAGAGTGATGAGG | |
| | | R | GTTTGAGTCGCCAATTCCCTTGTAGC | |
| | *LFY* | F | GAAGGGTTATCTGAGGAACCG | |
| | | R | TCCACTGACGTCAGCATTGG | |
| | *PP2A* | F | TATCGGATGACGATTCTTCGTGCAG | |
| | | R | GCTTGGTCGACTATCGGAATGAGAG | |

**Table S3. Sequences of oligonucleotide primers used in qRT-PCR analysis.**

| Type | Length | Nucleotides Sequences |
|---|---|---|
| ***pre-miR156a*** | 156bp | TGGGACAAAAGAAACGCAAAGAAAC**TGACAGAAGAGAGTGAGCAC**ACAAAAGCAATTT<br>GCATATGTTTATGTCCAAGCCGATCTCACTGCATTTGCTTCTCTTGCGTGCTCACTGC<br>TCTTTCTGTCAGATTCCGGTGCTGATCTCTTTGGCCTGTC |
| ***pre-miR156b*** | 173bp | GAGACTGAAGAAATGCAACAGAGAAAAC**TGACAGAAGAGAGTGAGCAC**ATGCAGGCAC<br>TGTTATGTCTCCATAACCTTGCGCGTGCGTGCTCACCTCTCTATCTGTCAGTTGCCTA<br>CCTCTAGCTTTCTCTCCCCTCTCTCTCTCTCTCTCTATCTCACACTAAAATCTGCTT |
| ***pre-miR156c*** | 276bp | CCATTTCTTTAGACCGATCTCTAATCACAGAATCTTTGTCGGTAAGATCATTGAGTCC<br>TGAGTTTTGTGGACAAATTTTAAGAGAAACGTAAAGAAAAC**TGACAGAAGAGAGTGAG**<br>**CAC**ACAAAGGCACTTTGCATGTTCGATGCATTTGCTTCTCTTGCGTGCTCACTGCTCT<br>ATCTGTCAGATTCCGGACCCGATTCAGTTTGGTCTGTTCATGTTTTCTTCTTTTAAAT<br>GTGTTCCCAAATCTCTCTCTCTTTATATCTCTATCTTGTGTTCT |
| ***pre-miR156d*** | 131bp | GTGGTTATATGTGTTGATGGGGAAAAAGAAGTTGACAGAAGAGAATGAGCACACAAAG<br>GGGAAGTTGTATATATAATAATTTGTATACGGTTGCTTTTGCGTGCTCACTCTCTTTC<br>TGTCATAACTTCATC |
| ***pre-miR156f*** | 134bp | GGATGGTTAGAGTGATGAGGAATTGATGG**TGACAGAAGAGAGTGAGCAC**ACATGGTGG<br>CTTTCTTGCATATTTGGATGTTTCATGCTTGAAGCTATGTGTGCTCACTCTCTCTCCG<br>TCACCTCTTCTCTCTATC |
| ***pre-miR156g*** | 132bp | CCAAAAGGTAAGTGAATAACTATGAGGAAGG**CGACAGAAGAGAGTGAGCAC**ACATGGC<br>TCCTTTTTCCAGCATGCTCATGCTCGAAAGCTCTGCGTGCTCACCCTCTTCTTGTCTC<br>CCTCTTGCTCTCCTTA |

**Table S4. Nucleotides sequences of miR156 precursors in *Arabis alpina* Pajares.** Red colored nucleotides indicate a target binding site of miR156s.
